# Concordant geographic and genetic structure revealed by genotyping‐by‐sequencing in a New Zealand marine isopod

**DOI:** 10.1002/ece3.6802

**Published:** 2020-12-02

**Authors:** William S. Pearman, Sarah J. Wells, Olin K. Silander, Nikki E. Freed, James Dale

**Affiliations:** ^1^ School of Natural and Computational Sciences Massey University Auckland New Zealand; ^2^ School of Environmental and Animal Sciences Unitec Institute of Technology Auckland New Zealand

**Keywords:** evolution, genetics, genomics, genotyping‐by‐sequencing, isolation‐by‐adaptation, isolation‐by‐distance, isopod, population, radseq

## Abstract

Population genetic structure in the marine environment can be influenced by life‐history traits such as developmental mode (biphasic, with distinct adult and larval morphology, and direct development, in which larvae resemble adults) or habitat specificity, as well as geography and selection. Developmental mode is thought to significantly influence dispersal, with direct developers expected to have much lower dispersal potential. However, this prediction can be complicated by the presence of geophysical barriers to dispersal. In this study, we use a panel of 8,020 SNPs to investigate population structure and biogeography over multiple spatial scales for a direct‐developing species, the New Zealand endemic marine isopod *Isocladus armatus*. Because our sampling range is intersected by two well‐known biogeographic barriers (the East Cape and the Cook Strait), our study provides an opportunity to understand how such barriers influence dispersal in direct developers. On a small spatial scale (20 km), gene flow between locations is extremely high, suggestive of an island model of migration. However, over larger spatial scales (600 km), populations exhibit a clear pattern of isolation‐by‐distance. Our results indicate that *I. armatus* exhibits significant migration across the hypothesized barriers and suggest that large‐scale ocean currents associated with these locations do not present a barrier to dispersal. Interestingly, we find evidence of a north‐south population genetic break occurring between Māhia and Wellington. While no known geophysical barrier is apparent in this area, it coincides with the location of a proposed border between bioregions. Analysis of loci under selection revealed that both isolation‐by‐distance and adaption may be contributing to the degree of population structure we have observed here. We conclude that developmental life history largely predicts dispersal in the intertidal isopod *I. armatus*. However, localized biogeographic processes can disrupt this expectation, and this may explain the potential meta‐population detected in the Auckland region.

## Graphical abstract

We show, for a New Zealand isopod, a strong relationship between geographic and genetic relationships. *Isocladus armatus* exhibits both isolation‐by‐distance and geographic concordance with a PCA. Unusually, we find evidence of high and ongoing gene flow across a well studied biogeographic break, which in other species hinders connectivity.

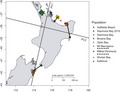

## 1 INTRODUCTION

A wide variety of factors act to determine genetic structure within populations of marine organisms. For example, variation in life‐history traits, such as habitat specificity or dispersal ability can result in different opportunities for gene flow. Population genetic structure can also arise as a consequence of the presence of biogeographic barriers such as ocean currents, land masses, or continental shelves limiting gene flow across the barrier. Alternatively, genetic structure can result from selection acting in different populations, for example, differential adaptation in heterogeneous environments (divergent selection).

In marine organisms, dispersal ability is highly influenced by developmental mode, which can be direct or biphasic. Biphasic species generally exhibit a pelagic larval stage, during which dispersal over large distances can occur via ocean currents. In contrast, direct developers have juveniles that resemble adults. Dispersal in most direct developers is achieved via relatively small‐scale mechanisms such as floating, rafting, creeping, or hopping (Winston, 2012).In these species, the stepping stone model of dispersal (where populations exchange migrants most frequently with sites that are close proximity) is the most common pattern of dispersal (Palumbi, 1994). This leads to a frequent isolation‐by‐distance pattern of population genetic variation in which genetic differentiation increases with geographic distance. This geographically limited dispersal of direct‐developing species predicts that they should exhibit greater population genetic structure relative to their biphasic counterparts (Ayre et al., 2009; McMillan et al., 1992; Pelc et al., 2009; Puritz et al., 2017; Waples, 1987). However, this is not true in all cases (Palumbi, 1994). For example, a comparative study on the phylogeography of Australian marine invertebrates showed no effect of a biogeographic barrier on genetic structure for two direct‐developing species, the banded periwinkle, *Austrolittorina unifasciata,* and the carpet sea star, *Meridiastra calcar* (Ayre et al., 2009), while the same barrier had a strong effect in six biphasic species (Ayre et al., 2009). Gene flow across this biogeographic barrier in these two direct developers was enabled by small patches of habitat across the barrier functioning as stepping stones for dispersal. This barrier consisted of a 300 km stretch of highly variable environmental conditions, alongside habitat and oceanographic discontinuity (Ayre et al., 2009). Thus, it has been proposed that habitat availability may be a better predictor of genetic structure than life history (Ayre et al., 2009).

Even within restricted taxonomic divisions, population genetic structure is not easily predicted from developmental life history. Marine isopods, like all peracarid crustaceans, are direct developers. Previous work has established that marine isopods often exhibit strong genetic structure over small spatial scales, on the order of tens of kilometers or less (e.g., *Idotea chelipes* (Jolly et al., 2003), *Austridotea lacustris* (McGaughran et al., 2006), and *Jaera albifrons* (Piertney & Carvalho, 1994)). This small‐scale structure is congruent with the hypothesis of reduced dispersal in direct developers and may be responsible for the widespread occurrence of multiple cryptic species of isopods (*Ligia* and *Tylos* spp.) on the Southern Californian coastline (Hurtado et al., 2010, 2013; Markow & Pfeiler, 2010). However, the mangrove boring isopod, *Sphaeroma terebrans*, is also a direct developer, but is widely distributed across both the Atlantic and Indian Oceans. The relationship between life history and population structure is complicated by the fact that multiple factors can act to influence population structuring. For example, Riginos et al. (2011) observed that, in fishes, both life‐history traits (i.e., egg type) and biogeography (i.e., biogeographic regions delineated on a range of contemporary and historical factors) were significant predictors of population structure. Furthermore, the New Zealand intertidal isopod *Limnoria*, exhibits population genetic structuring that is associated with both kelp genetic structuring, and large‐scale ocean currents in the sub‐Antarctic—suggestive of a strong influence of rafting on genetic structuring (Nikula et al., 2010). Thus, the specific mechanism of dispersal (e.g., rafting) can shape population structure, alongside dispersal mode (i.e., larval dispersal vs. direct development). Thus, it is clear that developmental life history alone is not a reliable predictor of population genetic structure.

Population genetic structure can also be influenced by geography (such as isolation‐by‐distance) or selection (such as isolation‐by‐adaptation). In particular, geographic distance has the potential to influence population structure, especially for coastally restricted direct‐developing species with purported limited dispersal (Hellberg, 1994; Palumbi, 2003). This can produce correlations between population genetic differentiation and geographic distance between populations (i.e., isolation‐by‐distance). However, selection often cannot be ruled out as a contributor to this correlation because geographic distance can also create environmental gradients over which selection can occur. For example, geographic distance over a latitudinal range can be associated with a temperature gradient, which may promote selection on certain loci. Disentangling the selective and nonselective causes of population structure can be achieved by examining neutral versus non‐neutral genetic variation (Kirk & Freeland, 2011). This is because greater genetic divergence at non‐neutral loci compared to neutral loci is predicted between populations experiencing divergent selection.

Additional work is required to elucidate the complex interplay between life history (dispersal potential and development mode), biogeography, and selection in determining population genetic structure. To shed light on the relative importance of these factors on population genetic structuring, we examined population structure across various spatial scales in a direct‐developing marine isopod endemic to New Zealand, *Isocladus armatus*. Isopods can be particularly informative for studying these questions in direct‐developing organisms, as their limited dispersal potential is expected to drive divergence between populations.

New Zealand presents an excellent opportunity to study marine isopods because it is a hotspot of marine isopod biodiversity (Bruce, 2009; Hurley & Jansen, 1977). Additionally, many New Zealand isopod species are abundant and easily sampled in intertidal zones across extensive geographic ranges (Bruce, 2009; Hurley & Jansen, 1977; Wells & Dale, 2018). Here we explore possible mechanisms responsible for maintaining this diversity using the intertidal isopod, *I. armatus*. This species exhibits several characteristics that make it an ideal candidate for study. First, populations are found in abundance on easily sampled semisheltered rocky shorelines. Second, they are found across a wide geographic range throughout the country (Jansen, 1971). Third, it is a highly color polymorphic species. This is interesting both from a natural history point of view, and because it is an easily identifiable trait that may be under strong selection, possibly affecting genetic diversity and population structure. Fourth, *I. armatus* is highly mobile, and has a strong swimming ability, often found swimming within the incoming tide (Jansen, 1971; Morton & Miller, 1973). This has the potential to either increase or decrease gene flow, as strong swimming ability may encourage dispersal, but may also help limit the tidal displacement of individuals. The mechanism of dispersal in *I. armatus* is not known. Although *I. armatus* is known to swim more in disturbed water, they will rapidly exit the water column and settle onto seaweed when available (Jansen, 1971). This behavior could facilitate their dispersal via rafting on dislodged seaweeds in the dynamic littoral zone, especially as many Sphaeromatid isopods, including those within *Isocladus*, are found in macroalgal holdfasts (Hurley & Jansen, 1977).

**FIGURE 1:**
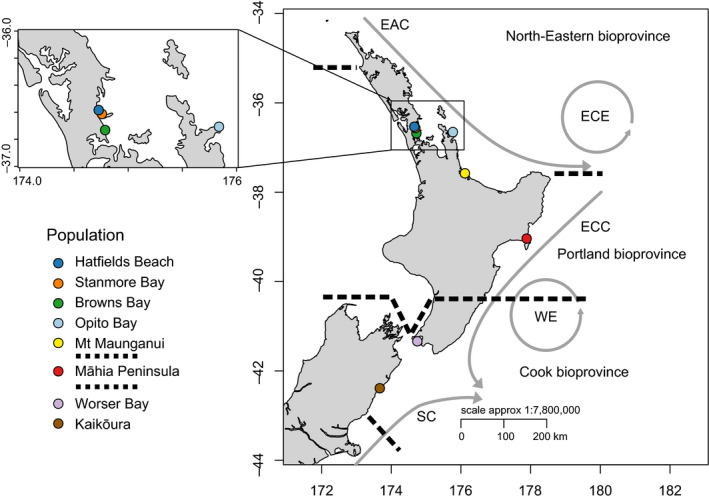
Map of sampling localities within New Zealand (colored dots). The prevailing ocean currents and biogeographic breaks proposed by Shears et al. (2008) are also indicated. Black dashed lines and unabbreviated labels indicate bioprovinces. Grey arrows indicate currents, abbreviated as: EAC (East Auckland Current), ECE (East Cape Eddy), ECC (East Cape Current), WE (Wairarapa Eddy), and SC (Southland Current)

A previous population genetic study of *I. armatus* found no significant population genetic structure between two sites separated by 11 km of coastline (Wells & Dale, 2018), but strong population genetic structure over a larger geographic scale (1,000 km). However, because no sampling was conducted at intermediate spatial scales, it is unclear at what scale this population genetic structure begins to break down. Furthermore, the stretch of coastline sampled in Wells and Dale (2018) is intersected by multiple biogeographic breaks such as the East Cape and the Cook Strait (Figure 1), and crosses multiple biogeographic regions. These biogeographic regions, defined by Shears et al. (2008) describe marine regions across New Zealand that exhibit discrete and identifiable differences in community assemblages and structure to each other. Recent evidence from a multispecies analysis of population genetic variation in New Zealand points to correspondence of the borders of these biogeographic regions with genetic structuring (Arranz Martinez, 2017). Thus, it is not well‐established how population genetic structure in this isopod species is affected by biogeographic barriers.

In order to shed light on these factors, we used genotyping‐by‐sequencing (GBS) to resolve population genetic structure in *I. armatus* for both neutral and non‐neutral loci. We sampled populations over a range of spatial scales and at locations intermediately located between previously sampled sites. These data allowed us to test (a) how population structure and dispersal is influenced by geographic distance, (b) whether known biogeographic barriers such as the East Cape and the Cook Strait influence gene flow and dispersal, and (c) whether there are divergent selective pressures among populations that are responsible for population genetic divergence.

## 2 METHODS

### 2.1 Sample collection

We collected specimens of *I. armatus* between May and July 2018, from around the North Island, New Zealand, from locations where *I. armatus* had previously been recorded (Hurley & Jansen, 1977; iNaturalist, n.d.). These sites were Stanmore Bay, Browns Bay, Opito Bay (Coromandel), Mt Maunganui, Māhia Peninsula, and Wellington (Figure 1). At each site we collected a minimum of 32 individuals. Because *I. armatus* is extremely color polymorphic, we opted to sample evenly across morph type to allow us to test for any influence of morph type on population structure. When possible, we collected specimens larger than 5 mm in order to provide enough tissue for DNA extraction (see Table A1 for details). We ensured that the maximum distance between individuals collected at any site did not exceed 30 m. We stored samples at −80°C in 100% ethanol until DNA extraction. For all analyses, here, we included previously collected samples from 2015 from Hatfield's Beach, Stanmore Bay (sampled again in 2018 for this study), and Kaikoura (see Wells and Dale (2018) for sampling methods) to increase the number of sampled individuals and the spatial sampling range. In addition, the two sampling events of Stanmore Bay were also used to explore any short‐term changes in allele frequencies within the population.

### 2.2 DNA extraction

We extracted DNA following a modified Qiagen DNEasy Blood and Tissue protocol from Wells and Dale (2018). Briefly, we used 178 µl of 0.5 M EDTA (pH 8) and 22 µl of 20% SDS in each extraction (rather than varying volumes by weight of tissue). Additionally, we eluted DNA from the spin column three times, using 50 µl of nuclease‐free water for each elution. We let the eluent sit on the column for 15 min before centrifugation for one minute at 7,000 rcf.

### 2.3 Data collection and processing

DNA samples were processed by Diversity Arrays Technology (DArT) Ltd using DArTseq, a genotyping‐by‐sequencing (GBS) approach. The restriction enzymes PstI and SphI were chosen for complexity reduction, the complete methodology for this approach is outlined in Wells and Dale (2018), but see (Kilian et al., 2012). DArT performs SNP calling using a proprietary pipeline. SNPs are only called if both homozygous and heterozygous genotypes can be identified (Wells & Dale, 2018).

We analyzed the dataset provided by Diversity Arrays Technology together with the data from Wells and Dale (2018). To ensure the datasets were compatible, we filtered each dataset separately based on the conditions described below using the R packages dartR (Gruber et al., 2018) and radiator (Gosselin, Lamothe, Devloo‐Delva, Grewe, 2020). We then used only the SNPs shared across both datasets for the remainder of the analyses.

We required SNPs to have a call rate ≥ 0.9 (i.e., a genotype was identified for at least 90% of individuals), a minor allele count of at least 3 (Linck & Battey, 2019; Rochette et al., 2019), observed heterozygosity > 0.5 (Hohenlohe et al., 2011), minimum mean depth of 5× and maximum mean depth of 50X (excessively high coverage may suggest duplicated genome elements which could confound further analyses) (Hohenlohe et al., 2011). If we found multiple SNPs on the same read, we removed the SNPs which had the lowest replicability (based on the number of technical replicates that resulted in the same allele being called). This step removes SNPs in clear linkage disequilibrium. We also removed SNPs that we inferred as being under strong selection. Non‐neutral SNPs are expected to exhibit different allele frequency spectra to neutral SNPs, and may bias inference of population demographics (Luikart et al., 2003). We identified SNPs under selection using BayeScan (Foll & Gaggiotti, 2008), with population as the grouping factor. SNPs exhibiting a *q*‐value of ≤ 0.05 were excluded from any further analyses.

While it is common practice to filter SNPs based on being in Hardy–Weinberg equilibrium (HWE) (Morin et al., 2009; Van Wyngaarden et al., 2017; Waples, 2015; Wells & Dale, 2018), this is not always the best practice. For example, STRUCTURE clusters individuals into populations in such a way to minimize Hardy–Weinberg disequilibrium overall (Pritchard et al., 2010). Implementing a HWE filter on SNPs can remove SNPs that are informative on population genetic structure. Thus, it may only be appropriate to remove SNPs that are either out of HWE in all populations, or out of HWE at an extremely restrictive significance level (this may be the result of selection, or genotyping errors). We opted for the former. Regardless, after all other filters were implemented, no SNPs remained that were out of HWE in all populations. Finally, we excluded one individual from Browns Bay from all analyses, as this sample had a very high number of SNPs missing across all positions (93%).

For some analyses, we separately analyzed the SNPs that were deemed under selection based on the BayeScan results. To do this, we performed the same filtering steps above, except that we did not filter on minor allele count or heterozygosity, and we removed SNPs that were considered neutral (*q*‐value ≥ 0.05) – this was because we wanted to retain any SNPs under putative selection, and both heterozygosity and minor allele count can signal non‐neutrality (Hernandez et al., 2019; Oleksyk et al., 2008).

### 2.4 Data analysis

We calculated population *F* statistics using StAMPP (Pembleton et al., 2013). We used pairwise *F*_st_ as the primary measure of population genetic differentiation (Whitlock, 2011). *p*‐values for the pairwise comparisons were adjusted for multiple comparisons using the Benjamini and Yekutieli correction in R (Benjamini & Yekutieli, 2001).

We conducted principal component analyses (PCA) using the R package adegenet (Jombart & Ahmed, 2011). This approach apportions genetic variation between individuals without any a priori assumptions of sampling location or reliance on a specific population model. Therefore, PCA is a useful first step in describing population structure. In order to understand the correspondence between the principal components and geography, we performed a Procrustes transformation of the first two principal components using MCMCpack in R (Martin et al., 2011). Procrustes transformations scale, stretch, and rotate the PCA in order to minimize the differences between two matrices (in this case, the difference between principal components and geographic coordinates).

We also examined population genetic structure using the Bayesian clustering approach implemented in the software STRUCTURE v.2.3.4 (Falush et al., 2003). We performed this analysis with all populations using the full set of neutral loci (including the repeated samples of Stanmore Bay). This method implements a model‐based clustering approach which probabilistically assigns individuals to one or more populations under an admixture model. These clustering analyses are particularly useful as they are naïve to sampling locations (thus avoiding the conflation of sampling locations and genetic populations) but incorporate population genetic models that can help describe population structure. For these analyses, we assumed an admixture model with correlated allele frequencies. This model assumes that individuals can have shared ancestry from one or more of *K* genetic clusters, rather than the no admixture model which assumes no shared ancestry between any of *K* genetic clusters. The admixture model is thus appropriate as we see clear instances of admixture in the PCA. We ran the Markov Chain Monte Carlo simulations with 100,000 iterations and a burn‐in of 50,000. We conducted ten replicates of each run and varied *K* from 2 to 9. We performed the final population inference by consolidating the results for each level of *K* in CLUMPP (Jakobsson & Rosenberg, 2007).

Additionally, we performed a separate STRUCTURE analysis on the Auckland populations with the implementation of the locprior model at a *K* of 3, in order to test for fine‐grain population structure within Auckland. Due to concerns regarding the inferences made when defining *K,* we chose to present a range of realistic values for *K* (Lawson et al., 2018; Pritchard et al., 2010; Verity & Nichols, 2016). In the case of hierarchically arranged populations, different values of *K* can provide different, but relevant, biological meaning (Hahn, 2019; Lawson et al., 2018; Pritchard et al., 2010). The assumption that there is a true single value of *K* is rarely correct and may lead to misinterpretations—especially in the absence of reliable demographic information, or where true discrete populations do not occur (Lawson et al., 2018; Meirmans, 2015). As a result of these situations, some authors have advocated for the presentation of multiple values of *K* and the use of penalized log likelihood for selection of *K* (Hubisz et al., 2009; Meirmans, 2015; Rosenberg et al., 2002).

In order to test whether the observed population genetic structure could be explained by an isolation‐by‐distance model, we tested for a correlation between genetic and geographic distances using standard and partial Mantel tests in the R package vegan (Oksanen et al., 2019). In a standard Mantel test we used a matrix of Slatkin's linearized *F*_st_ (transformed using 1/1 −* F*_st_ (Rousset, 1997)) as a measure of genetic differentiation, and an overwater distance matrix was used as an indicator of geographic distance. We calculated overwater distance using the marmap (Pante & Simon‐Bouhet, 2013) and fossil (Vavrek, 2011) R packages, finding the minimum distance between populations around the coast within a depth range of 150 m. For the partial Mantel test, we repeated the analysis above, but included a matrix of linearized pairwise non‐neutral *F*_st_ as a covariate. This analysis allows us to examine whether any signature of isolation‐by‐distance could be due to potential selection across environmental gradients preferentially affecting loci under selection, or the result of nonadaptive differentiation generated by geographic distance itself.

We conducted a hierarchical Analysis of Molecular Variance (AMOVA) using the R packages *ade4* (Dray & Dufour, 2007) and *poppr* (Kamvar et al., 2014). This approach partitions the variance in genetic data among and within each level of a predefined hierarchy (e.g., individuals, populations, or large geographically defined groups) (Excoffier et al., 1992). We tested the significance of the AMOVA by performing 1,000 random permutations using the R package *pegas* (Paradis, 2010). We partitioned the genetic variance according to the hierarchy of individuals, morphotype, sampling location, and then by the observed north‐south division found in our other analyses (among regions). We allow partitioning between colors because *I. armatus* is extremely color polymorphic and these colors may influence genetic variance, for example, if mating in frequency is affected by color polymorphism.

To test whether divergent selection in response to heterogeneous environments among populations (i.e., isolation‐by‐adaptation) could be occurring among diverse lineages of *I. armatus*, we repeated the AMOVA and *F*_st_ analyses for SNPs that were deemed to be under selection in our BayeScan analysis (Van Wyngaarden et al., 2017).

## 3 RESULTS

To examine population structure in *I. armatus* populations, we isolated 261 individuals distributed across eight populations across New Zealand (Figure 1). We obtained DArTseq SNP data for these 261 individuals, which identified 78,927 SNPs as being polymorphic. After stringent filtering, 8,020 SNPs were retained (Table A2).

All analyses showed clear evidence of population structure between most populations. We first conducted *F* statistics on this filtered SNP data to test for population differentiation. We found evidence of genetic differentiation even between geographically proximal populations. Pairwise *F*_st_ between Browns Bay and Stanmore Bay (separated by 18 km) was very low (*F*_st_ = 0.002; Table 1) but remained statistically significant after correction for multiple comparisons. *F*_st_ values generally increased with geographic distance. However, there was a sharp increase in genetic differentiation (from *F*_st_ values around or less than 0.1 to values around 0.35) for population comparisons that included populations south of Māhia Peninsula. Kaikoura and Māhia Peninsula (separated by 730 km) exhibited the greatest differentiation, with an *F*_st_ of 0.409 (Table 1). Surprisingly, the *F*_st_ observed between Hatfields Beach and Kaikoura was only 0.349, despite these two locations being separated by 1,317 km, almost twice the distance between Kaikoura and Māhia. Contrary to expectations, there was also no increase in *F*_st_ between Mt Maunganui and Māhia representative of the presence of a biogeographic barrier at East Cape.

**TABLE 1:** Pairwise comparisons of population *F*_st_ values

	Hatfields Beach 2015	Stanmore Bay 2015	Stanmore Bay 2018	Browns Bay 2018	Opito Bay 2018	Mt Maunganui 2018	Māhia Peninsula 2018	Wellington 2018
Stanmore Bay 2015	0 (*p* = 1)							
Stanmore Bay 2018	0 (*p* = 0.76)	0 (*p* = 1)						
Browns Bay 2018	0.001 (*p* = 0.005)	0.002	0.002					
Opito Bay 2018	0.037	0.036	0.036	0.036				
Mt Maunganui 2018	0.073	0.075	0.074	0.074	0.033			
Māhia Peninsula 2018	0.119	0.118	0.118	0.118	0.096	0.083		
Wellington 2018	0.338	0.338	0.343	0.34	0.363	0.382	0.402	
Kaikōura 2018	0.349	0.349	0.353	0.351	0.374	0.392	0.409	0.154

Note

There is evidence of weak genetic differentiation among some of the Auckland populations (Hatfields Beach and Stanmore Bay with Browns Bay) with a minimal significant *F*_st_ of 0.002. However, population structure between Wellington/Kaikoura and all other locations is strong, with a maximal *F*_st_ of 0.409 between Kaikōura and the Māhia Peninsula. *p*‐values were highly significant (*p* < .0001) and remained significant after adjustment for multiple comparisons, with the exception of the Auckland population comparisons with *p*‐values shown which were nonsignificant after this adjustment.

**FIGURE 2:**
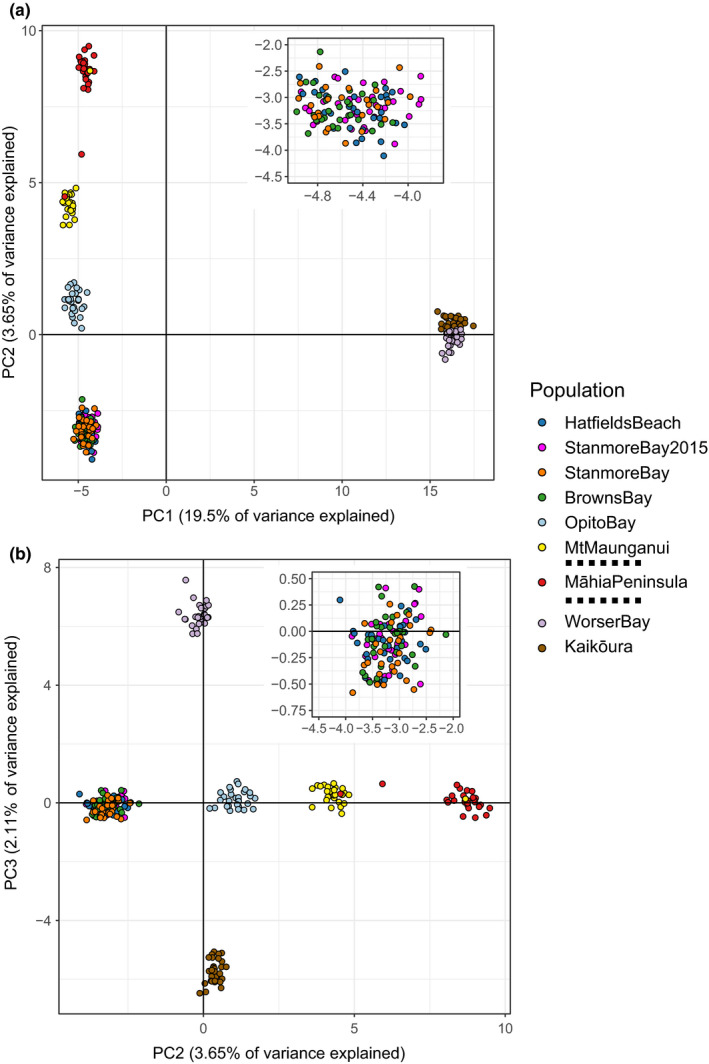
Principal component analysis (PCA) indicates strong location‐dependent population structure. In panel (a), PC1 (19.5% of the variance) largely differentiates the individuals in southern populations from those in the northern populations (i.e., Wellington and Kaikōura from the rest). PC2 (3.65% of the variance) differentiates between the northern populations. Finally, PC3 differentiates the populations within the southern group. Insets show the Auckland populations and indicate minimal evidence for structure within them. Three potential recent migrant individuals are apparent, two from the Māhia population (red points) to the Mt Maunganui population (yellow points), and one from the Mt Maunganui population to the Māhia population, visible in panel (b) within the red points. Dashed lines in the key indicate division among populations across bioprovinces

**FIGURE A1:**
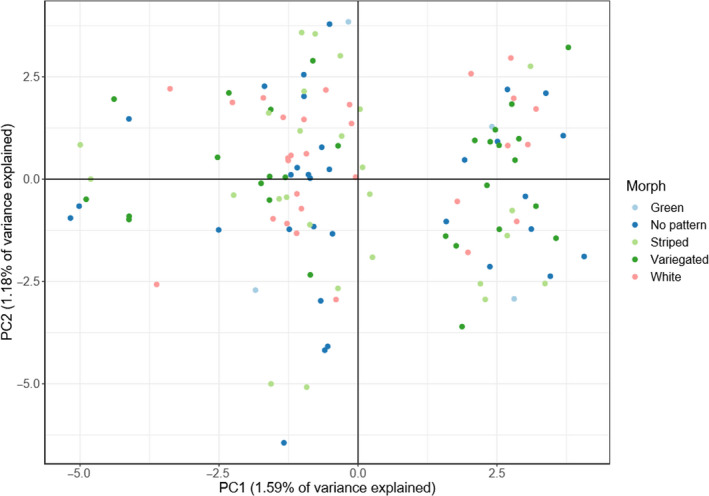
Principal Component Analysis of Auckland populations, colored by morphotype. No substructuring or stratifying effect of coloration is observed

Population structure was examined using principal component analysis, which identifies the combinations of SNPs that vary the most between individuals. We found that PC1 accounted for 19.5%, PC2 for 3.65%, and PC3 for 2.11% of the variance in SNP allele frequency (Figure 2a,b). PC1 primarily delineated the southern Wellington and Kaikōura populations from all other populations. PC2 primarily differentiated between the northern populations and revealed three potential cases of migration between Mt Maunganui and the Māhia Peninsula (Figure 2a). Finally, PC3 differentiated the two southernmost populations (Wellington and Kaikoura). All three Auckland populations clustered together, suggesting these individuals form either a single panmictic population, or a meta‐population. We found no difference between the temporal samples from Stanmore Bay (due to a complete overlap of the points representing the 2015 and 2018 samples in the PCA plot, Figure 2a), indicating lack of significant temporal variation in allele frequency. We also found no influence of color morph on genetic structure through PCA (Figure A1).

**FIGURE 3:**
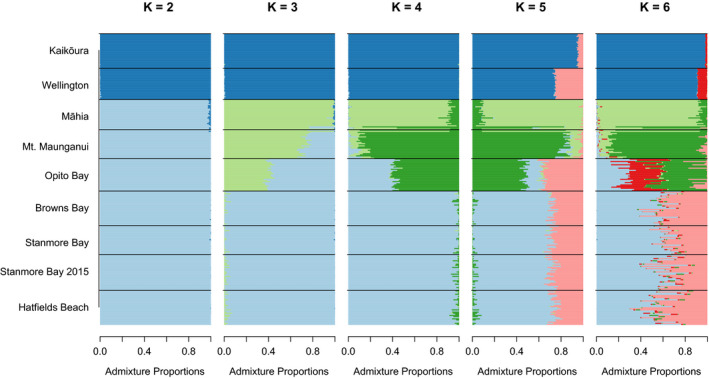
Admixture plots based on Bayesian clustering analyses generated in STRUCTURE. Each horizontal colored line represents an individual from the locality sampled (indicated on the left). Horizontal black lines designate the junction between population samples. Results are based on 10 replicate runs for each *K*. At *K* = 4 there are two individuals from Māhia with Mt. Maunganui‐like admixture profiles (bottom two bars in the Māhia samples), and one individual from Mt. Maunganui with a Māhia‐like admixture profile (top bar in the Mt. Maunganui samples)

**FIGURE A2:**
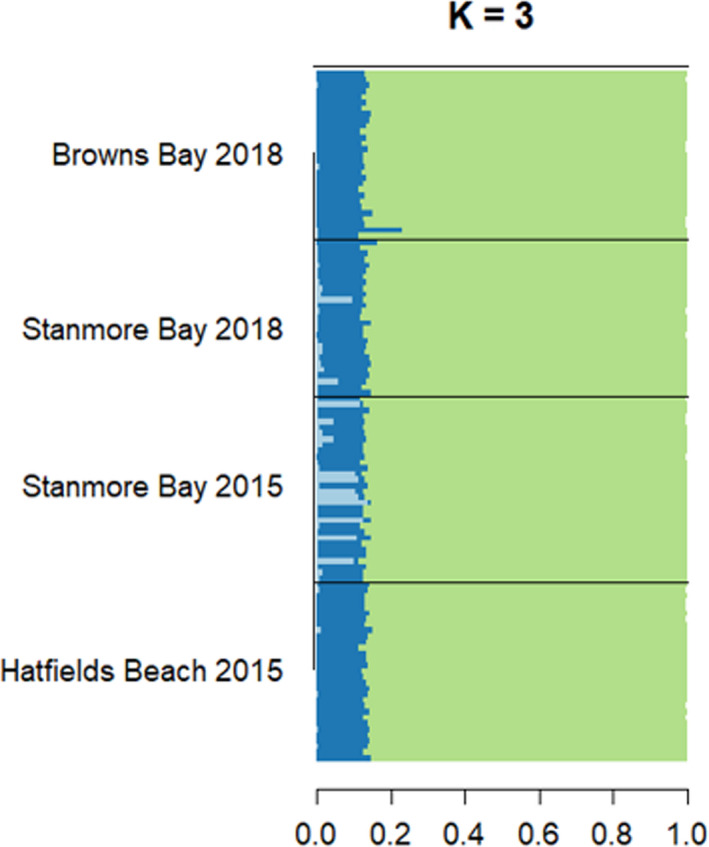
Admixture barplots generated through STRUCTURE and CLUMPP for a value of 3 for *K*. This analysis was done with the locprior model on just Auckland populations and was used to identify fine grain structure. Mean log likelihood is −4.6 × 10^5^ and variance is 8,324.2

The STRUCTURE analyses showed similar results to the PCA, with all individuals from the Auckland locations (Hatfield's Beach, Stanmore Bay, and Browns Bay) consistently clustering together, while Kaikōura and Wellington also always clustered together across all ranges of *K* (Figure 3). No additional fine‐grain structure within Auckland was observed when sampling location was incorporated into the analysis (through use of the locprior model) (Figure A2).

**FIGURE A3:**
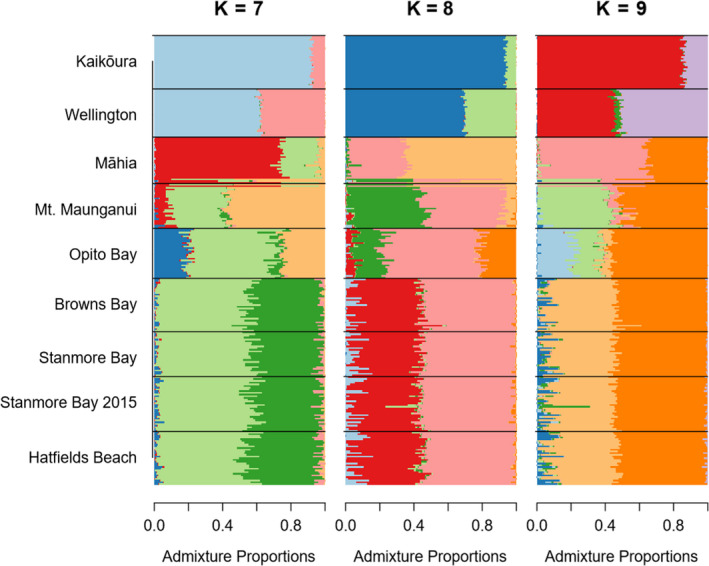
Admixture plots based on Bayesian clustering analyses generated in STRUCTURE for *K* 7–9. Relatively little structure is observed, and a much higher amount of noise is observed as *K* increases. In all instances, Kaikoura and Wellington retain a high degree of separation from other peoples, and Māhia also retains some distinctiveness

**FIGURE A4:**
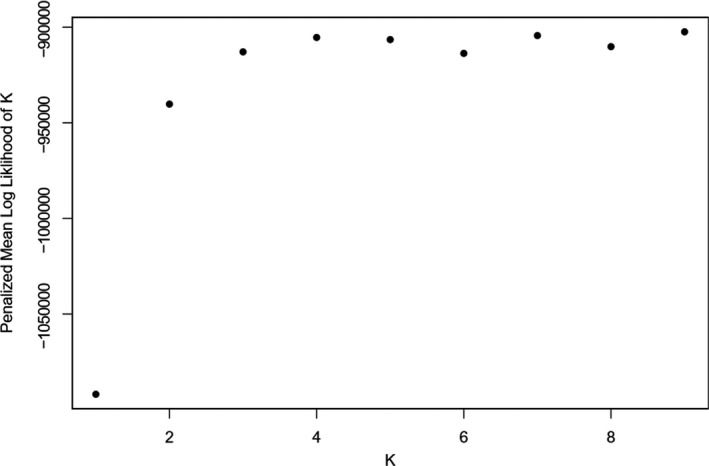
Mean log likelihood for STRUCTURE analyses penalized by variance. As *K* increased, the penalized log likelihood increases, but stops increasing at a *K* of 4—thus, we have primarily relied on a *K* of 4 for most results

With values of increasing *K*, additional population genetic structure became apparent. At *K* = 3, Māhia individuals formed a distinct cluster exhibiting high admixture with populations to the north (Mt. Maunganui and Opito Bay). Increasing *K* further suggested genetic admixture between all adjacent populations with the exception of Wellington and Māhia. With *K* = 4, there was clear evidence of two Māhia individuals with admixture profiles more similar to Mt. Maunganui individuals, and one Mt. Maunganui individual with a profile similar to Māhia individuals (Figure 3, bottom two individuals in the Māhia sample and top individual in the Maunganui sample, respectively). These correspond to the potential migrants identified in the PCA above. At *K* = 5 and *K* = 6, there was evidence of admixture between the northernmost populations (Hatfield's Beach, Stanmore Bay, and Browns Bay with Opito Bay) and the southernmost (Wellington and Kaikōura). However, at *K* = 7, Wellington and Kaikōura again clustered separately, with no admixture indicated with the northern populations. These results may be an artifact of overfitting which is also seen for greater values of *K* (Figure A3) (Evanno et al., 2005). Therefore, we rely on *K* = 4 as the most appropriate value of *K* in our discussion, based on penalized log likelihood (Figure A4) (Hubisz et al., 2009).

**FIGURE 4:**
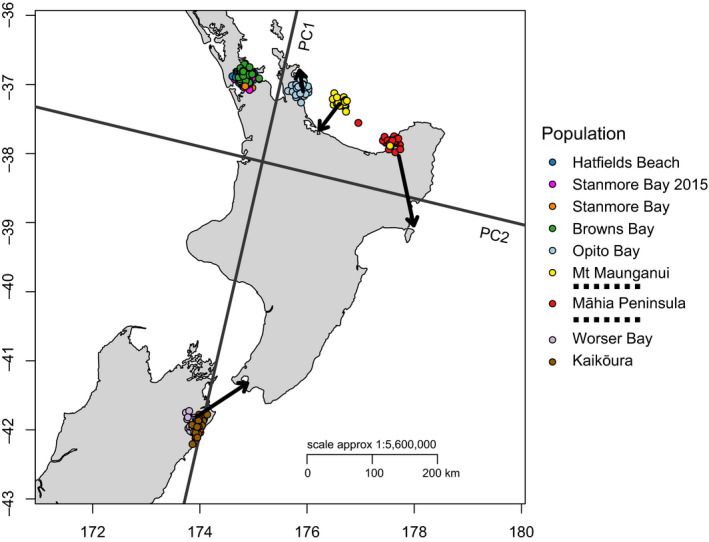
Procrustes transformation of PC1 versus PC2 onto geographic coordinates. The transformation indicates a strong correspondence between sampling location and genotype distance in principal component space. The arrows point to the location where a population is actually found, while clusters indicate their location in Procrustes‐transformed space. The bottom arrow indicates the location of the Wellington population (purple) only. Dashed lines in the key indicate division among populations across bioprovinces

**FIGURE 5:**
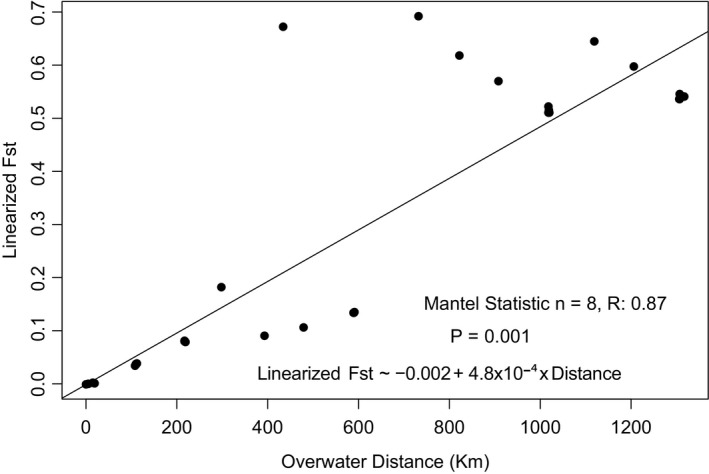
The positive correlation between overwater distance and Slatkin's linearized *F*_st_ suggests a pattern of population genetic structure due to isolation‐by‐distance. The black line indicates the slope of relationship for just the contrasts between the northern populations (points in the lower left quadrant). The equation represents the linear model used to generate the black line, predicting genetic distance from geographic distance

We next tested for a correlation between geographic and genetic distance. The Procrustes transformation of PC1 and PC2 revealed high correspondence between genetic distance and geographic distance, suggestive of an isolation‐by‐distance (IBD) effect (Figure 4). This was confirmed by the use of a Mantel test, which supported the hypothesis of IBD, revealing a significant positive correlation between Slatkin's linearized *F*_st_ and geographic overwater distance (Mantel test; *n* = 8, *r* = .87, *p* = 0.001; Figure 5). This strong positive relationship remained after the two southernmost and most genetically divergent populations (Kaikōura and Wellington) were excluded (Mantel test; *n* = 6; *r* = .97; *p* = 0.002). Finally, a partial Mantel test of *F*_st_ of neutral SNPs against distance, using a matrix of *F*_st_ of non‐neutral SNPs as a covariate, showed a significant correlation (partial Mantel test, *n* = 8, *r* = .77; *p* = 0.001), suggesting that this signature of IBD cannot be attributed solely to divergent selection operating across an environmental gradient and that the physical distance between populations is responsible for IBD, especially among the northern populations.

Finally, we also performed an analysis of molecular variance to allow for the partitioning of genetic variation among various hierarchical levels. This analysis confirmed the existence of strong genetic structure between the southern group (Wellington and Kaikōura) and the northern group. The greatest source of variation was within individuals (56.23%), and the second largest source of variation (28.91%) occurred between regions (Wellington/Kaikōura vs. all other populations, Table 2).

**TABLE 2:** AMOVA analysis of both neutral (*n* = 8,020) and non‐neutral (*n* = 392) SNPs shows population structure between populations and regions based on genetic variation, with regions accounting for the largest source of variation in non‐neutral SNPs

Source of variation	*df*	Percentage of variation in neutral SNPs (*p*‐value)	Percentage of variation in non‐neutral SNPs (*p*‐value)
Within individuals	261	56.23 (<0.001)	31.91 (<0.001)
Between individuals within morphotype	219	10.1 (<0.001)	5.48 (<0.001)
Between morphotype within populations	33	0.098 (0.2)	0 (0.81)
Between populations within region	7	4.65 (<0.001)	9.86 (<0.001)
Between regions	1	28.91 (0.028)	52.74 (0.026)

Note

We defined regions as the north group (all populations excluding Kaikōura and Wellington) and south group (Kaikōura and Wellington).

When an AMOVA was conducted on non‐neutral SNPs only, 52.74% of the total genetic variance occurred between regions. This between‐region variance was 23.8% greater than the between‐region variance identified using neutral SNPs, suggesting that selection may be acting to create a genetic barrier between these regions.

## 4 DISCUSSION

In this study, we quantified population genetic structure in a direct‐developing marine invertebrate across a wide range of spatial scales. By employing a GBS approach with thousands of genomic markers, we aimed to increase the resolution of the spatial scale at which population structure can be detected.

Despite our large dataset of 8,020 SNPs, there was minimal evidence of population structure of *I. armatus* within the Auckland region. These results, indicating low levels of population structure, are consistent with those from previous population genetic investigation in this species (Wells & Dale, 2018), as well as in some biphasically developing marine invertebrates in the Hauraki Gulf surrounding Auckland, such as the native New Zealand sea urchin *Evechinus chloroticus* (Nagel et al., 2015) and the invasive tunicate *Styela clava* (Goldstien et al., 2010). However, analysis of *F* statistics suggested weak differentiation between Browns Bay and the other Auckland populations (c. 20 km apart), although these values were very low relative to our other pairwise population comparisons of *F*_st_. Further finer‐scale sampling across the Auckland region would be required to determine whether weak population structuring truly begins to accumulate at this spatial scale.

Over larger spatial scales, *I. armatus* exhibited strong patterns of isolation‐by‐distance (IBD). The Procrustes‐transformed PCA indicated that the first two principal components were highly concordant with the geographic arrangement of populations. A Mantel test of the IBD hypothesis also indicated a strong and almost linear correlation between geographic distance and genetic distance within the northern populations, indicative of a stepping stone model of distribution.

We found evidence for unusually high admixture between Mt. Maunganui and Māhia Peninsula, despite their separation by a well‐known biogeographic barrier, the East Cape (Knox et al., 2018; Stevens & Hogg, 2004; Veale & Lavery, 2012). The strongest evidence for this is the presence of three individuals that appeared to be recent migrants between these populations. One individual from Māhia clustered with Mt Maunganui in the PCA, while the converse was the case for the second individual. The third individual was located between two clusters in the PCA. These three individuals also clustered with the alternate population or were highly admixed with it, in the STRUCTURE analysis, further supporting the migrant hypothesis. The intermediate genotype of one individual, as indicated by both the PCA and STRUCTURE analyses, argues against these results being due to sample contamination or a mix‐up. We propose that the admixture events that we have identified in our analyses may be the result of recent dispersal. One possible mechanism of how this could have occurred is from rafting events, where individuals can disperse between populations on floating debris (Baratti et al., 2011; Nikula et al., 2010). While rafting against prevailing currents may seem counter‐intuitive, Fraser et al. (2018) showed that wave‐driven surface currents such as Stokes drift explain the counter‐current dispersal of kelp rafts. *I. armatus* can be found on the same rocky coastlines as New Zealand kelps such as *Ecklonia radiata* and *Durvillaea antarctica,* and are known to cling to seaweeds in disturbed water (Jansen, 1971). Thus, rafting on kelp using Stokes drift could provide a mechanism of counter‐current dispersal in *I. armatus*.

While these results suggest that isolation‐by‐distance is responsible for small‐ to medium‐scale population structure in *I. armatus*, similar patterns of population structuring can also arise from a lack of migration‐genetic drift equilibrium within populations. For example, if a population expansion or migration event occurred recently in the past, then the observed genetic structure should be interpreted as a historical estimate of population genetic processes (Bohonak, 1999). Thus, the lack of population structure seen among the Auckland populations could be due to a migration‐drift disequilibrium that has arisen from a recent range expansion, rather than extensive gene flow between populations. This possibility could be further investigated by conducting population expansion tests on each population.

The hypothesized biogeographic break produced by the East Cape Eddy (Figure 1) has been shown to affect population structure in a range of species. This includes direct developers such as the anemone, *Actinia tenebrosa,* and two species of amphipods (Stevens & Hogg, 2004; Veale & Lavery, 2012), as well as biphasic species with larval stages, such as the pāua, *Haliotis iris* (Will et al., 2011) and the marine gastropod, *Buccinulum vittatum* (Gemmell et al., 2018). However, in contrast to these species, this break does not appear to strongly affect *I. armatus*, because *F*_st_ between Mt. Maunganui and Māhia was no greater than *F*_st_ between Mt. Maunganui and the Auckland area. In addition, PCA indicated that the Mt. Maunganui population was genetically intermediate between Māhia and Opito Bay, despite being geographically closer to Opito Bay. Finally, Bayesian clustering analyses showed genetic admixture between Māhia and Mt. Maunganui across all values of *K*, suggesting recent gene flow between these populations. The absence of a biogeographic break at East Cape could result from the existence of suitable patches of habitat across the coastline in this region, enabling greater success in migrant establishment, as proposed by Ayre et al. (2009). Previous reports of a biogeographic break in this region affecting direct developers have been based on either estuarine species (Stevens & Hogg, 2004), which may experience reduced habitat availability, or a sessile species whose limited motility may inhibit successful rafting (Veale & Lavery, 2012).

Our study does not support the existence of genetic barriers across either the Cook Strait (Goldstien et al., 2006) or the East Cape (Stevens & Hogg, 2004) because we observe unremarkable genetic differentiation between populations either side of these proposed barriers. Instead, we found a strong north‐south genetic disjunction between Māhia and Wellington in all analyses, with the largest *F*_st_ differences being between these two locations, and genetic clustering of the Wellington and Kaikōura populations. This north‐south break is congruent with the placement of a proposed border between bioregions in this area (Shears et al., 2008) (Figure 1). Shears et al. (2008) observed clear north‐south differences across both macroalgal and invertebrate community assemblages, but no clear differences between bioregions either side of East Cape. Indeed, this north‐south break appears to be broadly significant across marine invertebrate taxa, and for species with low dispersal potential is found directly south of Māhia (Arranz Martinez, 2017). Alternatively, this genetic barrier could be the result of environmental gradients imposing selective pressures on *I. armatus, that is,* isolation‐by‐adaptation through divergent selection (Nosil et al., 2009; Van Wyngaarden et al., 2017). This is supported by the observation that there was greater genetic variation between these regions in an AMOVA of non‐neutral SNPs compared to an AMOVA of neutral SNPs only. While the removal of non‐neutral SNPs should go some way to alleviating the effect of selective pressures in our analyses, genomic hitchhiking of linked SNPs may explain the large amount of variance between regions even in neutral loci (Feder et al., 2012; Feder et al., 2012; Nosil et al., 2009).

If divergent selection is occurring, it could result in *I. armatus* forming either a species complex, or divergent lineages undergoing a speciation‐with‐gene‐flow process (Feder, et al., 2012). Within New Zealand, divergent lineages of the brooding brittle star, *Amphipolis squamata*, have been associated with strong north‐south divergence, similar to our observations of *I. armatus* (Sponer & Roy, 2002). Cryptic species have also been frequently observed in isopods (Hurtado et al., 2016; Leese et al., 2008; Markow & Pfeiler, 2010), and the degree of genetic divergence between the northern and southern group in *I. armatus* is similar to that found between other divergent lineages of isopods based on mitochondrial DNA (Leese et al., 2008). The potential for a species complex, or lineages undergoing speciation, is further supported by the observation of an individual from Browns Bay (which was excluded from all analyses) that, despite appearing morphologically similar to *I. armatus*, lacked 93% of SNPs that were present in other samples. While missing data is a common feature of reduced representation datasets, excessively high missingness in GBS data has been associated with divisions between species rather than populations (Tripp et al., 2017).

## 5 CONCLUSION

*Isocladus armatus* exhibits high levels of gene flow across small spatial scales. However, at distances greater than 20 km the level of population structure is consistent with the expectation of reduced dispersal in direct‐developing species, and the presence of IBD. Interestingly, the strongest genetic break we observed was between the Māhia Peninsula and Wellington, with populations forming a clear northern and southern grouping on either side of this break. This was unexpected, as other well‐known biogeographic barriers—the East Cape and the Cook Strait—appeared to have little effect on population genetic structure. Our results suggest either a strong geophysical barrier to gene flow occurs between these regions, or that *I. armatus* represents divergent lineages undergoing speciation. Additional phylogeographic analysis and fine‐scale sampling across this genetic break would help determine whether the genetic divergence we observe is the result of genetic barriers to gene flow (such as selection), or the effects of a geophysical barrier that prevent dispersal in this region.

## CONFLICT OF INTEREST

The authors have no conflicts of interest to declare.

## AUTHOR CONTRIBUTIONs

**William S. Pearman:** Conceptualization (equal); data curation (equal); formal analysis (equal); investigation (equal); methodology (equal); visualization (lead); writing – original draft (lead); writing – review and editing (equal). **Sarah J. Wells:** Conceptualization (equal); data curation (equal); formal analysis (equal); funding acquisition (lead); investigation (equal); methodology (equal); writing – review and editing (equal). **Olin K. Silander:** Formal analysis (supporting); investigation (supporting); methodology (supporting); supervision (supporting); validation (supporting); writing – review and editing (supporting). **Nikki E. Freed:** Conceptualization (supporting); methodology (supporting); project administration (lead); resources (supporting); supervision (lead); writing – review and editing (supporting). **James Dale:** Conceptualization (equal); funding acquisition (equal); investigation (equal); methodology (equal); project administration (lead); resources (supporting); supervision (lead); writing – review and editing (equal).

## ETHICAL APPROVAL

No ethics approval or permits were required to conduct this research.

## Data Availability

Sequence data are available in the SRA under accession number: PRJNA643849, and associated metadata are deposited in the Ira Moana Project on GeOMe, under the expedition title “Isopod Sampling 2018”.

## 1

**TABLE A1:** Number of samples from each location grouped by sex and colour morphotype

Site	Sex	Striped	Unpatterned	Variegated	White	Other	Sum
Hatfields Beach 2015	Female	3	6	4	2	1	16
Hatfields 2015 2015	Male	0	4	4	4	3	15
Stanmore Bay 2015	Female	4	4	4	4	0	16
Stanmore Bay 2015	Male	4	4	4	4	0	16
Stanmore Bay	Female	4	4	3	4	0	15
Stanmore Bay	Male	3	3	2	3	0	11
Browns Bay	Female	4	4	4	4	0	16
Browns Bay	Male	4	4	4	4	0	16
Māhia Peninsula	Female	0	6	3	3	0	12
Māhia Peninsula	Male	2	9	4	0	0	15
Mt Maunganui	Female	1	3	3	4	0	11
Mt Maunganui	Male	4	4	4	3	0	15
Opito Bay	Female	4	6	1	2	0	13
Opito Bay	Male	3	4	4	5	0	16
Wellington	Female	0	4	5	5	0	14
Wellington	Male	0	5	5	4	0	14
Kaikōura 2015	Female	1	7	4	3	0	15
Kaikōura 2015	Male	1	5	4	0	5	15

Note

All sites are from 2018 except where explicitly stated.

**TABLE A2:** SNP filtering results and the number of SNPs retained after each filter

Condition	Number of SNPs remaining
Initial number	78,927
Present in Wells & Dale, 2018 and the current study, filtered separately	
Call Rate > 0.8	57,348
Depth > 3 and < 50	54,123
Replicability > 0.9	52,800
Monomorphic Loci	51,370
Secondary loci	31,848
Shared between datasets (*N* = 10,036 SNPs)	
Neutral loci	9,620
Call Rate > 0.9	8,334
Depth > 3 and < 50	8,334
Replicability > 0.95	8,334
Monomorphic loci	8,334
Secondary loci	8,334
Minor Allele Count > 3	8,037
Observed Heterozygosity < 0.5	8,020
Loci out of Hardy Weinberg Equilibrium within populations	4,552

Note

Datasets were first filtered separately and shared SNPs between the datasets were retained for the combined dataset. This was performed to minimize batch effects as a result of samples being sequenced at two different times. Additionally, the increased data output in the 2018 dataset (as a result of the inclusion of more individuals and populations) meant that almost twice as many SNPs were identified in this dataset, than in the 2015 dataset. By retaining shared SNPs the effect of this is minimized. Call rate refers to the proportion of individuals for which a genotype can be identified for the loci.

## References

[ece36802-bib-0001] Arranz Martinez, V. (2017). Connectivity among marine communities: a multi‐species approach to determining the major drivers of larval connection between populations of coastal species in New Zealand. PhD Thesis, ResearchSpace@Auckland.

[ece36802-bib-0002] Ayre, D. J. , Minchinton, T. E. , & Perrin, C. (2009). Does life history predict past and current connectivity for rocky intertidal invertebrates across a marine biogeographic barrier? Molecular Ecology, 18, 1887–1903. 10.1111/j.1365-294X.2009.04127.x 19434808

[ece36802-bib-0003] Baratti, M. , Filippelli, M. , & Messana, G. (2011). Complex genetic patterns in the mangrove wood‐borer *Sphaeroma terebrans* Bate, 1866 (Isopoda, Crustacea, Sphaeromatidae) generated by shoreline topography and rafting dispersal. Journal of Experimental Marine Biology and Ecology, 398, 73–82. 10.1016/j.jembe.2010.12.008

[ece36802-bib-0004] Benjamini, Y. , & Yekutieli, D. (2001). The control of the false discovery rate in multiple testing under dependency. Annals of Statistics, 29, 1165–1188. 10.1214/aos/1013699998

[ece36802-bib-0005] Bruce, N. L. (2009). The Marine Fauna of New Zealand: Isopoda, Aegidae (Crustacea). Auckland, New Zealand: National Institute of Water and Atmospheric Research.

[ece36802-bib-0006] Dray, S. , & Dufour, A.‐B. (2007). The ade4 package: Implementing the duality diagram for ecologists. Journal of Statistical Software, 22, 1–20.

[ece36802-bib-0007] Evanno, G. , Regnaut, S. , & Goudet, J. (2005). Detecting the number of clusters of individuals using the software STRUCTURE: A simulation study. Molecular Ecology, 14, 2611–2620. 10.1111/j.1365-294X.2005.02553.x 15969739

[ece36802-bib-0008] Excoffier, L. , Smouse, P. E. , & Quattro, J. M. (1992). Analysis of molecular variance inferred from metric distances among DNA haplotypes: Application to human mitochondrial DNA restriction data. Genetics, 131, 479–491. 1644282 10.1093/genetics/131.2.479 PMC1205020

[ece36802-bib-0009] Falush, D. , Stephens, M. , & Pritchard, J. K. (2003). Inference of population structure using multilocus genotype data: Linked loci and correlated allele frequencies. Genetics, 164, 1567–1587. 12930761 10.1093/genetics/164.4.1567 PMC1462648

[ece36802-bib-0010] Feder, J. L. , Egan, S. P. , & Nosil, P. (2012). The genomics of speciation‐with‐gene‐flow. Trends in Genetics, 28, 342–350. 10.1016/j.tig.2012.03.009 22520730

[ece36802-bib-0011] Feder, J. L. , Gejji, R. , Yeaman, S. , & Nosil, P. (2012). Establishment of new mutations under divergence and genome hitchhiking. Philosophical Transactions of the Royal Society B: Biological Sciences, 367, 461–474. 10.1098/rstb.2011.0256 PMC3233718 22201175

[ece36802-bib-0012] Foll, M. , & Gaggiotti, O. (2008). A genome‐scan method to identify selected loci appropriate for both dominant and codominant markers: A Bayesian perspective. Genetics, 180, 977–993. 10.1534/genetics.108.092221 18780740 PMC2567396

[ece36802-bib-0013] Fraser, C. I. , Morrison, A. K. , Hogg, A. M. , Macaya, E. C. , van Sebille, E. , Ryan, P. G. , & Waters, J. M. (2018). Antarctica's ecological isolation will be broken by storm‐driven dispersal and warming. Nature Climate Change, 8, 704–708. 10.1038/s41558-018-0209-7

[ece36802-bib-0014] Gemmell, M. R. , Trewick, S. A. , Crampton, J. S. , Vaux, F. , Hills, S. F. K. , Daly, E. E. , & Morgan‐Richards, M. (2018). Genetic structure and shell shape variation within a rocky shore whelk suggest both diverging and constraining selection with gene flow. Biological Journal of the Linnaean Society, 125, 827–843. 10.1093/biolinnean/bly142

[ece36802-bib-0015] Goldstien, S. , Schiel, D. R. , & Gemmell, N. J. (2006). Comparative phylogeography of coastal limpets across a marine disjunction in New Zealand. Molecular Ecology, 15(11), 3259–3268. 10.1111/j.1365-294X.2006.02977.x 16968269

[ece36802-bib-0016] Goldstien, S. J. , Schiel, D. R. , & Gemmell, N. J. (2010). Regional connectivity and coastal expansion: Differentiating pre‐border and post‐border vectors for the invasive tunicate *Styela clava* . Molecular Ecology, 19, 874–885. 20149095 10.1111/j.1365-294X.2010.04527.x

[ece36802-bib-0017] Gosselin, T. , Lamothe, M. , Devloo‐Delva, F. , Grewe, P. (2020). radiator: RADseq Data Exploration, Manipulation and Visualization using R. R package version 1.1.5. https://thierrygosselin.github.io/radiator/. 10.5281/zenodo.3687060

[ece36802-bib-0019] Gruber, B. , Unmack, P. J. , Berry, O. F. , & Georges, A. (2018). dartr: An r package to facilitate analysis of SNP data generated from reduced representation genome sequencing. Molecular Ecology Resources, 18, 691–699. 29266847 10.1111/1755-0998.12745

[ece36802-bib-0020] Hahn, M. (2019). Population structure In Sinauer (Ed.), Molecular population genetics (pp. 105–109). New York, NY: Oxford University Press.

[ece36802-bib-0021] Hellberg, M. E. (1994). Relationships between inferred levels of gene flow and geographic distance in a Philopatric Coral, *Balanophyllia elegans* . Evolution, 48, 1829–1854. 10.1111/j.1558-5646.1994.tb02218.x 28565162

[ece36802-bib-0022] Hernandez, R. D. , Uricchio, L. H. , Hartman, K. , Ye, C. , Dahl, A. , & Zaitlen, N. (2019). Ultra‐rare variants drive substantial cis‐heritability of human gene expression. Nature Genetics, 51, 1349–1355. 10.1038/s41588-019-0487-7 31477931 PMC6730564

[ece36802-bib-0023] Hohenlohe, P. A. , Amish, S. J. , Catchen, J. M. , Allendorf, F. W. , & Luikart, G. (2011). Next‐generation RAD sequencing identifies thousands of SNPs for assessing hybridization between rainbow and westslope cutthroat trout. Molecular Ecology Resources, 11, 117–122. 10.1111/j.1755-0998.2010.02967.x 21429168

[ece36802-bib-0024] Hubisz, M. J. , Falush, D. , Stephens, M. , & Pritchard, J. K. (2009). Inferring weak population structure with the assistance of sample group information. Molecular Ecology Resources, 9, 1322–1332. 10.1111/j.1755-0998.2009.02591.x 21564903 PMC3518025

[ece36802-bib-0025] Hurley, D. E. , & Jansen, K. P. (1977). The marine fauna of New Zealand: Family Sphaeromatidae (Crustacea Isopoda: Flabellifera). New Zealand Oceanographic Institute Memoir, 63, 1–80.

[ece36802-bib-0026] Hurtado, L. A. , Lee, E. J. , & Mateos, M. (2013). Contrasting phylogeography of sandy vs. rocky supralittoral isopods in the megadiverse and geologically dynamic Gulf of California and adjacent areas. PLoS One, 8, e67827 10.1371/journal.pone.0067827 23844103 PMC3699670

[ece36802-bib-0027] Hurtado, L. A. , Mateos, M. , Mattos, G. , Liu, S. , Haye, P. A. , & Paiva, P. C. (2016). Multiple transisthmian divergences, extensive cryptic diversity, occasional long‐distance dispersal, and biogeographic patterns in a marine coastal isopod with an amphi‐American distribution. Ecology and Evolution, 6, 7794–7808. 10.1002/ece3.2397 30128130 PMC6093162

[ece36802-bib-0028] Hurtado, L. A. , Mateos, M. , & Santamaria, C. A. (2010). Phylogeography of supralittoral rocky intertidal Ligia isopods in the Pacific region from Central California to Central Mexico. PLoS One, 5, e11633 10.1371/journal.pone.0011633 20657776 PMC2908127

[ece36802-bib-0029] iNaturalist . Available from https://www.inaturalist.org. Accessed May 2018.

[ece36802-bib-0030] Jakobsson, M. , & Rosenberg, N. A. (2007). CLUMPP: A cluster matching and permutation program for dealing with label switching and multimodality in analysis of population structure. Bioinformatics, 23, 1801–1806. 10.1093/bioinformatics/btm233 17485429

[ece36802-bib-0031] Jansen, K. P. (1971). Ecological studies on intertidal New Zealand Sphaeromatidae (Isopoda: Flabellifera). Marine Biology, 11, 262–285. 10.1007/BF00401274

[ece36802-bib-0032] Jolly, M. , Rogers, A. , & Sheader, M. (2003). Microgeographic generic variation of populations of *Idotea chelipes* (Crustacea: Isopoda) in lagoons of the southern English coast. Cahiers De Biologie Marine, 44, 319–327.

[ece36802-bib-0033] Jombart, T. , & Ahmed, I. (2011). adegenet 1.3‐1: New tools for the analysis of genome‐wide SNP data. Bioinformatics, 27, 3070–3071. 10.1093/bioinformatics/btr521 21926124 PMC3198581

[ece36802-bib-0034] Kamvar, Z. N. , Tabima, J. F. , & Grünwald, N. J. (2014). Poppr: An R package for genetic analysis of populations with clonal, partially clonal, and/or sexual reproduction. PeerJ, 2, e281 10.7717/peerj.281 24688859 PMC3961149

[ece36802-bib-0035] Kilian, A. , Wenzl, P. , Huttner, E. , Carling, J. , Xia, L. , Caig, V. , & Uszynski, G. (2012). Diversity arrays technology: A generic genome profiling technology on open platforms In Pompanon F. & Bonin A. (Eds.), Data production and analysis in population genomics, 888, (pp. 67–89). Totowa, NJ: Humana Press. https://doi.org/10.1007/978‐1‐61779‐870‐2_5 10.1007/978-1-61779-870-2_5 22665276

[ece36802-bib-0036] Kirk, H. , & Freeland, J. R. (2011). Applications and implications of neutral versus non‐neutral markers in molecular ecology. International Journal of Molecular Sciences, 12, 3966–3988. https://doi.org/10.3390/ijms12063966 21747718 10.3390/ijms12063966 PMC3131602

[ece36802-bib-0037] Knox, M. A. , Hogg, I. D. , & Pilditch, C. A. (2018). The role of vicariance and dispersal on New Zealand's estuarine biodiversity: The case of *Paracorophium* (Crustacea: Amphipoda). Biological Journal of the Linnean Society, 103(4), 863–874.

[ece36802-bib-0038] Lawson, D. J. , van Dorp, L. , & Falush, D. (2018). A tutorial on how not to over‐interpret STRUCTURE and ADMIXTURE bar plots. Nature Communications, 9, 1–11. 10.1038/s41467-018-05257-7 PMC6092366 30108219

[ece36802-bib-0039] Leese, F. , Kop, A. , Wägele, J. W. , & Held, C. (2008). Cryptic speciation in a benthic isopod from Patagonian and Falkland Island waters and the impact of glaciations on its population structure. Frontiers in Zoology, 5, 1–15. https://doi.org/10.1186/1742-9994-5-19 19099566 10.1186/1742-9994-5-19 PMC2644686

[ece36802-bib-0040] Linck, E. , & Battey, C. J. (2019). Minor allele frequency thresholds strongly affect population structure inference with genomic data sets. Molecular Ecology Resources, 19, 639–647. 10.1111/1755-0998.12995 30659755

[ece36802-bib-0041] Luikart, G. , England, P. R. , Tallmon, D. , Jordan, S. , & Taberlet, P. (2003). The power and promise of population genomics: From genotyping to genome typing. Nature Reviews Genetics, 4, 981–994. 10.1038/nrg1226 14631358

[ece36802-bib-0042] Markow, T. A. , & Pfeiler, E. (2010). Mitochondrial DNA evidence for deep genetic divergences in allopatric populations of the rocky intertidal isopod Ligia occidentalis from the eastern Pacific. Molecular Phylogenetics and Evolution, 56, 468–473. 10.1016/j.ympev.2009.12.002 20006723

[ece36802-bib-0043] Martin, A. , Quinn, K. , & Park, J. H. (2011). MCMCpack: Markov Chain Monte Carlo in R. Journal of Statistical Software, 42, 1–21.

[ece36802-bib-0044] McGaughran, A. , Hogg, I. D. , Stevens, M. I. , Lindsay Chadderton, W. , & Winterbourn, M. J. (2006). Genetic divergence of three freshwater isopod species from southern New Zealand. Journal of Biogeography, 33, 23–30. 10.1111/j.1365-2699.2005.01338.x

[ece36802-bib-0045] McMillan, W. O. , Raff, R. A. , & Palumbi, S. R. (1992). Population genetic consequences of developmental evolution in sea urchins (genus Heliocidaris). Evolution, 46, 1299–1312. 10.1111/j.1558-5646.1992.tb01125.x 28568989

[ece36802-bib-0046] Meirmans, P. G. (2015). Seven common mistakes in population genetics and how to avoid them. Molecular Ecology, 24, 3223–3231. 10.1111/mec.13243 25974103

[ece36802-bib-0047] Morin, P. A. , Leduc, R. G. , Archer, F. I. , Martien, K. K. , Huebinger, R. , Bickham, J. W. , & Taylor, B. L. (2009). Significant deviations from Hardy‐Weinberg equilibrium caused by low levels of microsatellite genotyping errors. Molecular Ecology Resources, 9, 498–504. 10.1111/j.1755-0998.2008.02502.x 21564679

[ece36802-bib-0048] Morton, J. E. , & Miller, M. (1973). The New Zealand Sea Shore. London, UK: Collins.

[ece36802-bib-0049] Nagel, M. M. , Sewell, M. A. , & Lavery, S. D. (2015). Differences in population connectivity of a benthic marine invertebrate *Evechinus chloroticus* (Echinodermata: Echinoidea) across large and small spatial scales. Conservation Genetics, 16, 965–978. 10.1007/s10592-015-0716-2

[ece36802-bib-0050] Nikula, R. , Fraser, C. I. , Spencer, H. G. , & Waters, J. M. (2010). Circumpolar dispersal by rafting in two subantarctic kelp‐dwelling crustaceans. Marine Ecology Progress Series, 405, 221–230. 10.3354/meps08523

[ece36802-bib-0051] Nosil, P. , Funk, D. J. , & Ortiz‐Barrientos, D. (2009). Divergent selection and heterogeneous genomic divergence. Molecular Ecology, 18, 375–402. 10.1111/j.1365-294X.2008.03946.x 19143936

[ece36802-bib-0052] Oksanen, J. , Blanchet, F. G. , Friendly, M. , Kindt, R. , Legendre, P. , McGlinn, D. , … & Wagner, H. (2019). vegan: Community Ecology Package. R package version 2.5‐3. Vienna: R Foundation for Statistical Computing https://CRAN.R‐project.org/package=vegan

[ece36802-bib-0053] Oleksyk, T. K. , Zhao, K. , De La Vega, F. M. , Gilbert, D. A. , O'Brien, S. J. , & Smith, M. W. (2008). Identifying selected regions from heterozygosity and divergence using a light‐coverage genomic dataset from two human populations. PLoS One, 3, e1712 10.1371/journal.pone.0001712 18320033 PMC2248624

[ece36802-bib-0054] Palumbi, S. R. (1994). Genetic divergence, reproductive isolation, and marine speciation. Annual Review of Ecology and Systematics, 25, 547–572. 10.1146/annurev.es.25.110194.002555

[ece36802-bib-0055] Palumbi, S. R. (2003). Population genetics, demographic connectivity, and the design of marine reserves. Ecological Applications, 13, 146–158. 10.1890/1051-0761(2003)013[0146:PGDCAT]2.0.CO;2

[ece36802-bib-0056] Pante, E. , & Simon‐Bouhet, B. (2013). marmap: A package for importing, plotting and analyzing bathymetric and topographic data in R. PLoS One, 8, e73051 10.1371/journal.pone.0073051 24019892 PMC3760912

[ece36802-bib-0057] Paradis, E. (2010). pegas: An R package for population genetics with an integrated–modular approach. Bioinformatics, 26, 419–420. 10.1093/bioinformatics/btp696 20080509

[ece36802-bib-0058] Pelc, R. A. , Warner, R. R. , & Gaines, S. D. (2009). Geographical patterns of genetic structure in marine species with contrasting life histories. Journal of Biogeography, 36, 1881–1890. 10.1111/j.1365-2699.2009.02138.x

[ece36802-bib-0059] Pembleton, L. W. , Cogan, N. O. I. , & Forster, J. W. (2013). StAMPP: An R package for calculation of genetic differentiation and structure of mixed‐ploidy level populations. Molecular Ecology Resources, 13, 946–952. 10.1111/1755-0998.12129 23738873

[ece36802-bib-0060] Piertney, S. B. , & Carvalho, G. R. (1994). Microgeographic genetic differentiation in the intertidal isopod Jaera albifrons Leach. I. Spatial distribution of allozyme variation. Proceedings of the Royal Society B: Biological Sciences, 256, 195–201.

[ece36802-bib-0061] Pritchard, J. K. , Wen, W. , & Falush, D. (2010). Documentation for STRUCTURE software: Version 2.3.3. http://web.stanford.edu/group/pritchardlab/software/structure_v.2.3.1/documentation.pdf

[ece36802-bib-0062] Puritz, J. B. , Keever, C. C. , Addison, J. A. , Barbosa, S. S. , Byrne, M. , Hart, M. W. , & Toonen, R. J. (2017). Life‐history predicts past and present population connectivity in two sympatric sea stars. Ecology and Evolution, 7, 3916–3930. 10.1002/ece3.2938 28616188 PMC5468144

[ece36802-bib-0063] Riginos, C. , Douglas, K. E. , Jin, Y. , Shanahan, D. F. , & Treml, E. A. (2011). Effects of geography and life history traits on genetic differentiation in benthic marine fishes. Ecography, 34, 566–575. 10.1111/j.1600-0587.2010.06511.x

[ece36802-bib-0064] Rochette, N. C. , Rivera‐Colón, A. G. , & Catchen, J. M. (2019). Stacks 2: Analytical methods for paired‐end sequencing improve RADseq‐based population genomics. Molecular Ecology, 28(21), 4737–4754. 10.1111/mec.15253 31550391

[ece36802-bib-0065] Rosenberg, N. A. , Pritchard, J. K. , Weber, J. L. , Cann, H. M. , Kidd, K. K. , Zhivotovsky, L. A. , & Feldman, M. W. (2002). Genetic structure of human populations. Science, 298, 2381–2385. 10.1126/science.1078311 12493913

[ece36802-bib-0066] Rousset, F. (1997). Genetic differentiation and estimation of gene flow from F‐statistics under isolation by distance. Genetics, 145, 1219–1228. 9093870 10.1093/genetics/145.4.1219 PMC1207888

[ece36802-bib-0067] Shears, N. T. , Smith, F. , Babcock, R. C. , Duffy, C. A. J. , & Villouta, E. (2008). Evaluation of biogeographic classification schemes for conservation planning: Application to New Zealand's coastal marine environment. Conservation Biology, 22, 467–481. 10.1111/j.1523-1739.2008.00882.x 18294299

[ece36802-bib-0068] Sponer, R. , & Roy, M. S. (2002). Phylogeographic analysis of the brooding brittle star *Amphipholis squamata* (Echinodermata) along the coast of New Zealand reveals high cryptic genetic variation. Evolution, 56, 1954–1967. 12449482 10.1111/j.0014-3820.2002.tb00121.x

[ece36802-bib-0069] Stevens, M. I. , & Hogg, I. D. (2004). Population genetic structure of New Zealand's endemic corophiid amphipods: Evidence for allopatric speciation. Biological Journal of the Linnaean Society, 81, 119–133. 10.1111/j.1095-8312.2004.00270.x

[ece36802-bib-0070] Tripp, E. A. , Tsai, Y.‐H.‐E. , Zhuang, Y. , & Dexter, K. G. (2017). RADseq dataset with 90% missing data fully resolves recent radiation of Petalidium (Acanthaceae) in the ultra‐arid deserts of Namibia. Ecology and Evolution, 7, 7920–7936. 29043045 10.1002/ece3.3274 PMC5632676

[ece36802-bib-0071] Van Wyngaarden, M. , Snelgrove, P. V. R. , DiBacco, C. , Hamilton, L. C. , Rodríguez‐Ezpeleta, N. , Jeffery, N. W. , & Bradbury, I. R. (2017). Identifying patterns of dispersal, connectivity and selection in the sea scallop, *Placopecten magellanicus*, using RADseq‐derived SNPs. Evolutionary Applications, 10, 102–117. 28035239 10.1111/eva.12432 PMC5192885

[ece36802-bib-0072] Vavrek, M. J. (2011). Fossil: Palaeoecological and palaeogeographical analysis tools. Palaeontologia Electronica, 14, 16.

[ece36802-bib-0073] Veale, A. J. , & Lavery, S. D. (2012). The population genetic structure of the waratah anemone (*Actinia tenebrosa*) around New Zealand. New Zealand Journal of Marine and Freshwater Research, 46, 523–536.

[ece36802-bib-0074] Verity, R. , & Nichols, R. A. (2016). Estimating the number of subpopulations (K) in structured populations. Genetics, 203, 1827–1839. 27317680 10.1534/genetics.115.180992 PMC4981280

[ece36802-bib-0075] Waples, R. S. (1987). A multispecies approach to the analysis of gene flow in marine shore fishes. Evolution, 41, 385–400. 10.2307/2409146 28568763

[ece36802-bib-0076] Waples, R. S. (2015). Testing for Hardy‐Weinberg proportions: Have we lost the plot? Journal of Heredity, 106, 1–19. 10.1093/jhered/esu062 25425676

[ece36802-bib-0077] Wells, S. J. , & Dale, J. (2018). Contrasting gene flow at different spatial scales revealed by genotyping‐by‐sequencing in *Isocladus armatus*, a massively colour polymorphic New Zealand marine isopod. PeerJ, 6, e5462 10.7717/peerj.5462 30155361 PMC6109376

[ece36802-bib-0078] Whitlock, M. C. (2011). *G*′_ST_ and D do not replace *F* _ST_ . Molecular Ecology, 20, 1083–1091. 10.1111/j.1365-294X.2010.04996.x 21375616

[ece36802-bib-0079] Will, M. , Hale, M. L. , Schiel, D. R. , & Gemmell, N. J. (2011). Low to moderate levels of genetic differentiation detected across the distribution of the New Zealand abalone, *Haliotis iris* . Marine Biology, 158, 1417–1429. 10.1007/s00227-011-1659-x

[ece36802-bib-0080] Winston, J. E. (2012). Dispersal in marine organisms without a pelagic larval phase. Integrative and Comparative Biology, 52, 447–457. 10.1093/icb/ics040 22505589

